# Is the Student-Centered Learning Style More Effective Than the Teacher-Student Double-Centered Learning Style in Improving Reading Performance?

**DOI:** 10.3389/fpsyg.2019.02630

**Published:** 2019-11-27

**Authors:** Yang Dong, Sammy Xiaoying Wu, Weisha Wang, Shuna Peng

**Affiliations:** ^1^Department of Social and Behavioural Sciences, City University of Hong Kong, Kowloon, Hong Kong; ^2^Department of Curriculum and Instruction, The Education University of Hong Kong, Tai Po, Hong Kong; ^3^Faculty of Business, Law and Art, Southampton Business School, University of Southampton, Southampton, United Kingdom; ^4^Faculty of Education and Science, Jiaying University, Meizhou, China

**Keywords:** learning style, teaching-learning style, reading research, reading comprehension, intervention

## Abstract

Appropriate learning styles enhance the academic performance of students. This research compares and contrasts the popular effect of teacher-student double centered learning style (TSDCLS) and student-centered learning style (SCLS) on one’s reading, including reading comprehension, inference, main idea abstraction, and reading anxiety. One hundred and fifty one students in grade 4 from three groups (two experimental groups and one control group) participated in the experiment with 18 weeks’ reading comprehension training. The results showed that, first, both learning styles contributed to students’ reading comprehension, inference, main idea abstraction, and reading anxiety. Second, the TSDCLS contributed more to reading anxiety, and the SCLS contributed more to reading comprehension. Both learning styles had similar effects on inference and main idea abstraction. From the correlation test, excluding the correlation between SCLS and reading anxiety which was not significant, all other effects were significant. These findings are discussed along with implications and ideas for future research.

## Introduction

Reading comprehension is an ability to gain meaning from the given text through the interaction between word decoding process and background knowledge application (Cain et al., 2004; Ahmed et al., 2016). Reading comprehension has received considerable attention as the ultimate goal for reading is to draw a mental image from the given text. The schema in linguistic learning (referred to herein as *linguistic schema*) plays a fundamental role in reading comprehension through literacy knowledge search in readers’ knowledge bases. To be more specific, in reading comprehension, linguistic schema works on the reading process through cue retrieval (e.g., vocabulary meaning) from the mental knowledge structure to interpret and integrate selected new information into the existing knowledge base (e.g., Nassaji, 2002). In reading comprehension, besides general comprehension ability, the other relevant abilities (e.g., inference and main idea abstract) and reading affect (e.g., reading anxiety) also determine the process of reading comprehension (Rai et al., 2015; Silva and Cain, 2015; Stanley et al., 2018).

Previous studies have confirmed that appropriate teaching-learning style enhances students’ academic performance (Komarraju et al., 2011; Huang et al., 2012), while there remains a general paucity of research comparing the different effects of each teaching-learning style on reading comprehension and the determining factors. Specifically, the effect of different teaching-learning styles on reading performance is a long way from being known. The correlations between different teaching-learning styles and reading factors (e.g., reading comprehension, reading anxiety, inference, and main idea) await discovery.

## Literature Review

### Linguistic Schema on Reading Process

Linguistic schema is an individual knowledge base for the reading comprehension process which activates background knowledge from schemata to support reading problem-solving (Freebody and Anderson, 1983; Hebert et al., 2016). It mainly works on cognitive reading abilities and cognitive affect (Cain et al., 2004; Sorrell and Brown, 2018). As for cognitive reading abilities, past studies showed that linguistic schema enhances readers’ first language (L1) reading comprehension abilities (e.g., inference, main idea abstraction) through meaning-based interpretations of the printed text (e.g., Cain et al., 2004).

### Teaching Style

Teaching style refers to a belief which teachers used in pedagogy explanation and knowledge transfer to students (Grasha and Yangarber-Hicks, 2000; Hsieh et al., 2011; Prescott, 2014). Under the guidance of self-determination theory (Ryan and Deci, 2000), teachers’ teaching performance was impacted by competence, autonomy, and relatedness. Extensive studies showed autonomy-supportive and controlling practices directly impacted teachers’ teaching style development (e.g., Balaguer et al., 2018; Codina et al., 2018; Collie et al., 2019). Both autonomy-supportive and controlling practices reflected teachers’ awareness of supporting students’ empowerment on knowledge acquisition, controlling action on feeling, and thinking. Therefore, the current study selected the autonomy-supportive and controlling practices as the framework for teaching style investigation. Moreover, extensive evidences showed teachers’ teaching style usually worked with students’ learning style, if teaching style matched learning style, students would achieve the best learning results than those whose teaching-learning style did not match due to decreased students’ academic anxiety, learning motivation, and task inattentiveness (Naimie et al., 2010; Chen et al., 2011; Bartholomew et al., 2018). In general, based on self-determination theory (Ryan and Deci, 2000), teaching style could be further divided into teacher-centered style and learner-centered style (Edmunds et al., 2008; Tessier et al., 2010; Haerens et al., 2015). Teacher-centered style refers to a teachers’ domain teaching approach in which the knowledge transfer was from the teacher to students directly; teacher played the decision-maker role, which determined learning process and designed the learning environment (Opdenakker and Van Damme, 2006; Kahl and Venette, 2010; Vasileva-Stojanovska et al., 2015). The learner-centered style reflected students who had high involvement in knowledge acquisition with which the teacher played a facilitative role (Özyurt and Özyurt, 2015; Rogowsky et al., 2015; Bernard et al., 2017). Past studies showed learner-centered style had advantages in increasing students’ deep understanding on knowledge acquisition (Lin, 2015; Hanewicz et al., 2017; Yamagata, 2018), learning motivation (McCombs et al., 2008; Polly and Hannafin, 2010), and improved critical thinking abilities (Ernst and Monroe, 2004; Cornelius-White, 2007; Şendağ and Odabaşı, 2009). In addition, past studies mainly investigated the effect of teaching-learning style in math, science, or engineering, a few studies examined the effect in the reading field. Therefore, the current study investigated the learner-centered style and investigated the effect on reading factors.

### Impact of Different Learning Styles on Reading Comprehension

Two main learner-centered styles drew great attention in the existing literature but still attract debate in the context of reading comprehension: SCLS and TSDCLS (Souvignier and Mokhlesgerami, 2006; Rance-Roney, 2010; Law, 2014). Past studies showed that the differences between two learning styles on academic performance mainly come from two factors: teachers’ instruction and students’ self-regulated learning. Although these two factors work[ed] cooperatively, they still reflect two distinct bodies in the learning process.

#### Teachers’ Instruction

Teachers’ instruction worked effectively on students’ reading comprehension through explicit guidance on key information or the application of reading strategies (Rance-Roney, 2010; Law, 2014). Some researchers claimed that teachers’ direct guidance (i.e., providing explicit instructions on selected reading task) were more effective than implicit instruction (i.e., pure interactive discussion between students) (Dole et al., 1991, 1996). For example, Dole et al. (1996) reported that students with direct guidance from the teacher performed better than those who undertook SCLS in narrative and expository texts.

#### Students’ Self-Regulated Learning

There are two key factors to initiate and maintain readers’ self-learning; cognitive control and motivational control (Boekaerts, 1999). On the one hand, researchers claimed that the provision of external structures to control cognitive and motivational regulation was effective in enhancing reading abilities during text comprehension (e.g., Boekaerts, 1999; Pintrich, 2000). For example, Souvignier and Mokhlesgerami (2006) reported that students under motivation control performed better in reading comprehension strategy application than the control group did. In a similar vein, previous studies identified that cognitive control enhanced readers’ performance in main idea abstraction and comprehension strategies’ application (Lau, 2012; Lau and Chen, 2013). On the other hand, many researchers showed that the explicit instruction in self-regulated learning may reduce the beneficial effects on students’ learning (e.g., Dignath et al., 2008; Dignath and Büttner, 2008). In two meta-analyses, Dignath et al. (2008) found that risk may be involved when teachers tailor self-regulated instruction; this is because teachers may over-control readers’ cognitive process and motivational process (Waeytens et al., 2002), and such intervention may reduce the effectiveness of self-regulated learning on students’ learning behaviors. There is no doubt that learner effect and teacher effect are both important in teaching-learning cycles. However, which learning style is more effective in enhancing reading comprehension and reading factors remains to be identified.

### Reading Anxiety

In the case of cognitive affect response, reading anxiety mainly reflects the emotional experience in reading (Saito et al., 1999). Reading anxiety refers to a distinct complex of self-perceptions, behaviors, and affective feelings related to text reading activity (Saito et al., 1999; Lu and Liu, 2015). Reading anxiety has a close relationship with reading comprehension. High reading anxiety inhibits reading performance and passage recall (Sellers, 2000; Rai et al., 2015). Different learning styles bring different learning experiences for the readers. For example, the teacher-centered learning style may leave learners feeling bored or disinterested in class (Schaefer and Zygmont, 2003; Lee and Hannafin, 2016). However, the effect of the learner-centered style (e.g., TSDCLS and SCLS) on reading anxiety is still unclear. Therefore, research on which learning style is better to reduce students’ reading anxiety is timely.

### Main Idea Abstraction

Main idea abstraction refers to an ability to abstract the mental message from the given test, which is the ultimate goal of reading comprehension. The main idea has a close relationship with reading performance, which in turn determines the reading process (e.g., reading speed) and how the readers can abstract/select the proper reading strategies from their knowledge bases (Hebert et al., 2018; Stevens et al., 2019). Previous studies confirmed that main idea abstraction is a key factor of executive function in reading comprehension. Usually, a higher main idea abstraction ability predicts higher reading performance. Learning style is essentially a habit that is adopted during the reading learning process, how readers’ learning style impacts the ability to develop the main idea remains unknown.

### Inference

Inference is a key ability in reading, which reflects readers’ ability to take in vocabulary knowledge and grammar knowledge in sentence reading comprehension (Cain et al., 2004, 2004). Inference is another important ability in reading executive function. Past researchers tried to improve readers’ inference ability to enhance reading comprehension (e.g., Bensoussan and Laufer, 1984), and studies confirmed that self-internal reading factors (e.g., vocabulary knowledge, reading strategies) predicted inference (Cromley and Azevedo, 2007; Ahmed et al., 2016). Learning style, as an external factor of learning, determined the learning process; however, it remains to be established whether the learning style has a positive impact on inference.

### The Current Study

There are two main aims of this research. The first is to compare and contrast the effectiveness of both SCLS and TSDCLS in enhancing readers’ reading experience, which includes overall reading comprehension performance, specific reading abilities (e.g., main idea abstraction and inference), and reading anxiety. Second, the research examines the relationship between each learning style as well as reading comprehension, inference ability, main idea, and reading anxiety.

## Method

### Participants

Participants for the research were randomly selected from three Grade 4 classes. Of these classes, all students come from low socio-economic status families and the three selected schools were in suburban districts in China. In previous learning, all students’ learning curricula followed standard instructions designed and approved by China’s Ministry of Education. All students were randomly divided into different classes after they finished the registration procedure for the school program. Consent was obtained from students and their parents before the formers’ participation in the research. No participants have received any form of reading ability training. In total, 151 students with no special education needs and from low socio-economic status families were randomly recruited and randomly allocated to three groups. About 49 (25 boys and 24 girls, mean_age_ = 10.12 years old, SD_age_ = 0.61) participated in the TSDCLS group, and 52 students (26 boys and 26 girls, mean_age_ = 9.98 years old, SD_age_ = 0.79) participated in SCLS group. Finally, 50 students (28 boys and 22 girls, mean_age_ = 9.82 years old, SD_age_ = 0.67) formed the control group.

### Measures

To examine the effectiveness of SCLS and TSDCLS in enhancing reading comprehension and reading factors, inference ability and main idea abstraction ability from the normal reading comprehension (NRC); NRC tests were used to measure students’ reading performance in primary school. The measurement details are listed in the following paragraph.

#### Normal Reading Comprehension

We examined students’ NRC reading abilities through three selected narrative passages (version 2015, version 2016, and version 2017), which were widely used, well-established, and with both high reliability (Cronbach’s *α*-coefficients yielded 0.90) and validity translation tests, which were applied to measure the Chinese children’s NRC reading ability in primary school from 2015 to 2017 (Xia, 2013; Zhang, 2018). Each reading comprehension test had similar difficulty indicators (e.g., correct answer rate in all samples around China was 60%), and the maximum score for each reading battery was 30 with 12 items. Three items for the main idea had a maximum score of six, and four items (e.g., what is the target word meaning in the passage) for inference had the total maximum score of eight (two scores for each). One item for the detailed search was awarded a score of four, and the remaining four items had a total score of 12. The length of each passage was around 900 words, a total of around 2,700 words for all three reading comprehension tests. Version 2015 reading comprehension test was used for pre-test, version 2016 reading comprehension test was used for post-test, and version 2017 reading comprehension test was used for the delayed test.

#### Reading Anxiety

Students’ reading anxiety was assessed by the Chinese Language Reading Anxiety Scale (CLRAS), which was developed by Zhao (2013); the original scale was developed by Saito et al. (1999). The CLRAS contains 20 items on a 5-point Likert scale ranging from “*strongly disagree*” to “*strongly agree*.” An example item is: “When reading a Chinese passage, I get nervous and confused when I don’t understand each word.” The scale assessed factors that may contribute to reading anxiety and considered students’ perceptions of the difficulties they encounter. A high score would indicate high levels of anxiety. The maximum mean score of the scale was 5 and Cronbach’s *α* for the scale was 0.90.

### Procedure

#### Research Design

We applied a quasi-experimental design to measure students’ performance in pretest, post-test, and lasting effect (hereafter, we called delayed post-test in the current study). First, we collected the consent forms from potential students and randomly selected students from three classes as our participants. Second, we randomly assigned a research design to each class. Random allocation resulted in 49 students in the TSDCLS group and 52 students in the SCLS group. The remaining 50 students were assigned to the control group. The control group received regular classroom instruction during the reading comprehension program training. The three classes were taught by one of the training tutors, who is also working as a part-time teacher in that school. All expectation teaching outcomes followed the curriculum outline design, which was approved by the Ministry of Education in China for both primary school and secondary school learning periods. Third, to control for irrelevant learning effects (e.g., NRC familiarity learning effect), we selected classical reading comprehension (CRC) for the study. The content structure of CRC is similar to straightforward poems, short works, and fresh but with lively literacy information (Wang, 2016). Specifically, all CRC vocabulary has at least two different meanings: *phonetic radical* meaning and *semantic radical* meaning (Shu and Anderson, 1997; Chen et al., 2016). Past studies in Chinese reading comprehension confirmed that CRC was a useful predictor of reading performance (Shu and Anderson, 1997; Wang, 2017). For CRC learning materials, normally, students begin to learn CRC at grade 5 of primary school, which means that, at the time of this study, participants had not done any CRC tasks in normal school test or school learning. The difference between CRC and NRC is that most CRC texts are narrative passages, the average length of each sentence of NRC is longer than that of CRC, and the meaning of each character is unique in NRC. Both TSDCLS and SCLS students were required to learn 21 CRC passages from the textbooks. The total training duration was from late February to late June (18 weeks).

In terms of reading performance examination, the pre-test was held in late February. Before the experiment started, all participants received reading comprehension tests (2015 version) and questionnaires regarding reading anxiety and inference. Regarding the post-test, the 2016 version of the reading comprehension test was used, with the same reading anxiety survey and inference tests. At the delayed post-test, the 2017 version of the reading comprehension test was used, and with the same reading anxiety survey and inference tests.

#### Training Materials

Training materials comprise three components. Component 1 is a vocabulary list that listed all vocabulary meanings in CRC with higher frequency and the vocabulary that students were required to know from the textbook teaching guidance. For example, “举(ju3)”: meaning 1, “并列 (equal effect),” “举案齐眉 (husband and wife treat each other with respect)”; and meaning 2, “全 (all),” “举国同庆(Nationwide celebration).” Component 2 has 21 passages from the school textbook, which the students were required to learn during the training program. All learning passages are narrative passages, telling a story. For example, “邹忌讽齐王纳谏 (Zou Ji’s Advice to the King).” In each passage, a modern language translation in direct translation style is given under each sentence, which means that we provided each character’s meaning in the second line of the given sentence. In addition, we highlighted the points of difficulty (e.g., grammatical knowledge and vocabulary knowledge) in the initial instructions guide. Component 3 is the after-school exercise, where all items were selected from the textbook. The exercise included main idea abstraction, inference sentence meaning, and detailed information search.

#### Training Procedure

The major difference between the TSDCLS group and SCLS group is that those students in TSDCLS had more interaction with teachers, which followed the guidance of the TSDCLS (Rogowsky et al., 2015). That is, in each lesson, teachers and students learn the given materials together. The teacher tries to get interaction with students on each kind of knowledge transfer. Specifically, the interaction between teacher and students mainly reflects in heuristic problem solving and knowledge recall through exercise, the average frequency of the interaction on each knowledge item was over four times.

For the SCLS group, at the beginning of each class, the teacher reminded students to read the guidance instructions carefully first and then asked students to learn the given passage, which was followed by the SCLS (Dignath et al., 2008). The teacher would only instruct students if students encountered difficulties and raised their hands. If more than three students had the same question, the teacher explained the knowledge being sought. In each training session, the teacher started class with encouragement on students’ self-learning, which mainly used self-questioning, self-regulated learning, and self-problem solving. The average interaction frequency during the learning period was one time only for time instruction and asked students to move their eyes to exercises to continue learning. All learning materials in the TSDCLS and SCLS group were the same and included the direct translation in modern language, the difficult points, the characters’ different meanings recall notice, grammatical function, and so on.

For the control group, except the material of guidance instructions, all three components’ training materials were provided to control group’s students at the same learning period.

## Results

### Descriptive Analysis and Comparison Reading Performance From Three Groups

For scores calculation, regarding the main idea test, we used items’ scores from the NRC. Then, we compared each reading factor performance in pre-test, post-test, and delayed post-test contexts.

Repeated ANOVA and four-way ANOVA were used for data analysis. From Table 1, at pre-test, all three groups’ students achieved similar performance in NRC (*F* = 0.30, *p* > 0.05), inference (*F* = 0.15, *p* > 0.05), main idea abstraction (*F* = 0.11, *p* > 0.05), and reading anxiety (*F* = 0.49, *p* > 0.05). At post-test, three groups students performed significant differences in NRC (*F* = 289.56, *p* < 0.001), inference (*F* = 285.05, *p* < 0.001), main idea abstraction (*F* = 112.63, *p* < 0.001), and reading anxiety (*F* = 49.89, *p* < 0.001). Specifically, regarding NRC, SCLS students performed significant higher scores than TSDCLS students (Mean difference = 1.00, *p* < 0.001), TSDCLS students performed significant higher score than control group (Mean difference = 3.80, *p* < 0.001). Regarding inference, TSDCLS students had similar scores with SCLS students (Mean difference = 0.09, *p* > 0.05), control group students had significant lower score with TSDCLS (Mean difference = 2.45, *p* < 0.001) and SCLS students (Mean difference = 2.36, *p* < 0.001). Regarding main idea abstraction, TSDCLS students performed similar score with SCLS students (Mean difference = 0.02, *p* > 0.05), control group students performed significant lower score with TSDCLS students (Mean difference = 1.86, *p* < 0.001) and SCLS students (Mean difference = 1.84, *p* < 0.001). Regarding reading anxiety, TSDCLS students had significant lower score than SCLS students (Mean difference = −0.79, *p* < 0.001), SCLS students performed significant lower score than control group students (Mean difference = −0.85, *p* < 0.001).

**Table 1:** Comparison means and standard deviations for three rounds of data collection from three different groups.

		NRC		Infer		Main idea		Reading anxiety	
		Mean	SD	Mean	SD	Mean	SD	Mean	SD
Pre-test	T-S	16.98	1.75	2.96	0.61	4.59	0.50	4.06	0.78
	S-S	16.62	1.75	2.92	0.74	4.38	0.49	3.90	0.82
	Con	17.12	1.60	3.18	0.77	4.52	0.51	3.88	0.85
	*F*	0.30		0.15		0.11		0.49	
Post-test	T-S	21.94	1.18	5.57	0.50	6.96	0.68	2.33	0.69
	S-S	22.94	0.70	5.48	0.51	6.94	0.67	3.12	0.92
	Con	18.14	1.23	3.12	0.72	5.1	0.79	3.96	0.81
	*F*	289.56^***^		285.05^***^		112.63^***^		49.89^***^	
Delayed post-test	T-S	22.35	0.97	5.41	0.50	7.16	0.69	2.29	0.84
	S-S	22.88	0.70	5.44	0.50	7.13	0.74	3.23	0.88
	Con	18.96	1.95	4.10	1.28	5.50	1.04	3.88	0.66
	*F*	131.71^***^		41.38^***^		65.15^***^		49.77^***^	

*T-S, TSDCLS; S-S, SCLS; Con, Control group; Infer, Inference ability*.

*** *p < 0.001*.

At delayed post-test, three group students performed significant differences in NRC (*F* = 131.71, *p* < 0.001), inference (*F* = 41.38, *p* < 0.001), main idea abstraction (*F* = 65.15, *p* < 0.001), and reading anxiety (*F* = 49.77, *p* < 0.001). Specifically, SCLS students performed significant higher score than TSDCLS students in NRC (Mean difference = 0.54, *p* < 0.05), TSDCLS students performed significant higher score than control group students (Mean difference = 3.39, *p* < 0.001). Regarding inference, TSDCLS students had similar score with SCLS students (Mean difference = 0.04, *p* > 0.05), control group students had significant lower score than TSDCLS students (Mean difference = 1.31, *p* < 0.001) and SCLS students (Mean difference = 1.34, *p* < 0.001). Regarding main idea abstraction, TSDCLS students got similar score with SCLS students (Mean difference = 0.03, *p* > 0.05). Control group students had significant lower score with TSDCLS students (Mean difference = 1.66, *p* < 0.001) and SCLS students (Mean difference = 1.64, *p* < 0.001). Regarding reading anxiety, TSDCLS students had significant lower reading anxiety than SCLS students (Mean difference = −0.95, *p* < 0.001), SCLS students had significant lower reading anxiety than control group students (Mean difference = −0.85, *p* < 0.001).

### The Effect of Both Teacher-Student Double Centered Learning Style and Student-Centered Learning Style on Reading and Reading Factors

To further explore the effect of both TSDCLS and SCLS on reading and reading factors, we created two sub-variables for learning styles and examined the effect on reading comprehension and reading factors.

From Table 2, in both the post-test and delayed post-test, the TSDCLS had significant positive correlations with NRC (*r*_pre-test_ = 0.27, *p* < 0.001; *r*_post-test_ = 0.30, *p* < 0.001), inference (*r*_pre-test_ = 0.46, *p* < 0.001; *r*_post-test_ = 0.28, *p* < 0.001), main idea (*r*_pre-test_ = 0.38, *p* < 0.001; *r*_post-test_ = 0.34, *p* < 0.001), and reading anxiety (*r*_pre-test_ = −0.54, *p* < 0.001; *r*_post-test_ = −0.58, *p* < 0.001), while for the SCLS, the SCLS had significant positive correlations with NRC (*r*_pre-test_ = 0.60, *p* < 0.001; *r*_post-test_ = 0.49, *p* < 0.001), inference (*r*_pre-test_ = 0.43, *p* < 0.001; *r*_post-test_ = 0.32, *p* < 0.001), and main idea (*r*_pre-test_ = 0.39, *p* < 0.001; *r*_post-test_ = 0.34, *p* < 0.001), but the correlation between SCLS and reading anxiety was not significant (*r*_pre-test_ = −0.02, *p* > 0.05; *r*_post-test_ = −0.07, *p* > 0.05).

**Table 2:** The effect of learning styles on reading and reading factors.

		NRC	Inference	Main idea	Reading anxiety
Post-test	T-S	0.27^***^	0.46^***^	0.38^***^	−0.54^***^
	S-S	0.60^***^	0.43^***^	0.39^***^	−0.02
Delayed post-test	T-S	0.30^***^	0.28^***^	0.34^***^	−0.58^***^
	S-S	0.49^***^	0.32^***^	0.34^***^	0.07

*T-S, TSDCLS; S-S, SCLS*;

*** *p < 0.001*.

## Discussion

We examined the effect of both TSDCLS and SCLS on reading comprehension and relevant reading factors (reading anxiety, main idea abstraction, and inference). The results showed that, firstly, the two experimental groups performed better than the control group in reading comprehension and three selected reading factors. Between TSDCLS and SCLS, the TSDCLS performed better in reading anxiety than the SCLS. However, the SCLS performed better in NRC than the TSDCLS. As for inference ability and main idea abstraction, two experimental groups had similar performance. Secondly, the TSDCLS had a significant positive correlation with reading comprehension and three selected reading factors, in which the results were consistent with the comparison test. However, the SCLS did not have a significant correlation with reading anxiety. We discussed this phenomenon later.

### The Advantage of Teacher-Student Double Centered Learning Style

Reading anxiety depends on an individual’s perceptions of a threatening situation and their abilities in handling this threat (Frenzel et al., 2016; Marsh et al., 2017). Students following the TSDCLS performed with less reading anxiety than those using the SCLS, which is consistent with those studies that indicated the positive effect of teachers’ support on students’ reading anxiety (Pratt and Savoy-Levine, 1998). Firstly, as Beck’s team (1985) suggested, a lower level of reading anxiety may come from less threatening learning situations, which means that, in the TSDCLS, the teacher’s guidance could be regarded as supportive information for assisting learning. Second, the TSDCLS provides a clear teacher-directed learning track, which could facilitate students to coordinate a goal-directed strategy, thus minimizing threatening emotions and maximizing safety (Beck, 1985). In the current study, in the TSDCLS, the teacher could guide students on how to learn CRC effectively and this teacher-directed learning track reduced students’ threatening experience on CRC learning. In addition, dialog interaction encourages students to solve reading questions at the time they occur, and teachers could immediately provide feedback. Third, the interactive situation may create a decentralized pattern of power, which may encourage students to have more incentive to engage in, and confidence in, knowledge search (Mezirow, 2000). Thus, the threatening emotions might be eased and the teacher’s support may gradually boost their safety awareness.

### The Advantage of the Student-Centered Learning Style

Better performance in the NRC test could be explained by two reasons. Firstly, the depth of processing theory shows that deeper encoding facilitates better memory retrieval compared with shallow encoding (e.g., Craik and Lockhart, 1972). In the current study, in the SCLS, with less teachers’ guidance, students were forced to make more effort to attain deeper and more levels of elaborating and associating the reading materials with their knowledge retrieval during training, which means they have more comprehensive understanding of reading knowledge (e.g., sentence structure, vocabulary meaning). In this case, they find it easier to retrieve more needed knowledge on a NRC task due to deeper encoding of memory cues. For example, when students need to accomplish a NRC task, they may recall target knowledge (e.g., phrases’ meaning) faster than students, who are exposed to the TSDCLS. Secondly, in the SCLS, students have more problem-solving opportunities to think about how to deal with reading tasks effectively, which means they may cultivate more useful reading strategies for reading comprehension practice. Specifically, with guided questions, students might be more likely to engage in retroactive elaborating on and encoding of learning materials where they tend to frequently regulate their learning strategies to use previously learned similar knowledge to tackle the problems at hand.

### Similar Effect Between Teacher-Student Double Centered Learning Style and Student-Centered Learning Style

#### Main Idea

The findings of similar abilities in main idea abstraction in both TSDCLS and SCLS groups were partially consistent with previous studies (e.g., Stoeger et al., 2014), but contrasting with the findings of Mason’s (2004) study, which proved the preference of the SCLS over the TSDCLS in teaching main idea abilities. The equal effect of finding main ideas observed in the current study might be related to the involvement of similar probes (i.e. verbal and written prompts) in the TSDCLS and the SCLS, respectively. Morrow (1985) managed to establish that students’ abilities in retelling stories were facilitated when they were guided with verbal prompts. The explicit guidance in both TSDCLS and SCLS groups may facilitate students to pick out relevant information in supporting the summarization. This result informed the ability of main idea abstraction could be learned through the appropriate track of guidance, no matter the teacher-directed learning or students’ self-learning based on explicit instruction guidance. Under the correct track of guidance, students could increase the ability of main idea abstraction.

#### Inference

As for the equal effect of inferences in both groups, according to the direct and inferential mediation (DIME) model, background knowledge is a significant component for enacting inferences. Prior studies also proved that different kinds of background knowledge about the texts resulted in higher inference scores (e.g., Vidal-Abarca et al., 2000). In the current study, in the beginning, from the test and survey, students in both TSDCLS and SCLS groups had similar background knowledge in NRC. As a result, there might be a possibility that similar storage of background knowledge has equal effects on their inferencing abilities in both TSDCLS and SCLS groups. That is to say, the amount of background knowledge acquisition on reading comprehension was similar between TSDCLS and SCLS through training materials.

### Other Effects From the Student-Centered Learning Style on Reading Anxiety

Results showed that the SCLS did not have a significant correlation with reading anxiety. However, the reading anxiety score decreased significantly at post-test and delayed post-test. The reason may be due to the students’ feelings of freedom when exposed to SCLS. In particular, during the CRC training, the reader increased other abilities, which decreased threatening feelings when they faced reading comprehension. The reason why the correlation between SCLS and reading anxiety was insignificant might be the compensation or interaction effect with other reading comprehension factors (e.g., word reading and metalinguistic knowledge), resulting in the insignificant correlation.

### Limitations and Future Direction

Several limitations of this study should be stated. First, due to the cognitive ability of primary school students, we only measured the effect of learning style on narrative reading comprehension, while other types of reading text such as descriptive reading comprehension and argumentative reading comprehension need further exploration. Second, the current study examined the TSDCLS and SCLS, which are popular nowadays in teaching, while for the teacher-centered learning style, the effect may be different from the learner-centered style (e.g., TSDCLS and SCLS). Third, to the best of our knowledge, there is no standardized and independent scale for inference ability and main idea abstraction measurement. Therefore, for future studies, there is great potential to design a standardized research scale. In addition, this study only investigated students’ general reading anxiety. Previous studies showed that anxiety could be categorized into “trait” and “state” anxiety (e.g., Nelson et al., 2015; Waechter and Stolz, 2015). Different types of anxiety have different effects on individuals’ executive function (e.g., working memory, inference, and main idea); thus leading to different reading performance efficiencies (e.g., Hadwin et al., 2005). Therefore, it is promising to develop scales eliciting different types of reading anxiety. Next, the correlation between SCLS and reading anxiety was not significant, the potential compensation or interaction effect with other reading factors requires further investigation. The factors that decreased reading anxiety are still unknown. Lastly, the current study only investigated two perspectives’ (autonomy-supportive and controlling practices) effects on teaching-learning style; for other perspectives’, effects such as competences and relatedness should be further explored in the future.

### Implications

The current study contributes to the correlation between TSDCLS and SCLS and reading comprehension from cognitive processing and affect. First, the SCLS with questions-probing learning materials could further strengthen reading ability development in previously-learned knowledge with some amount of learning foundation. The SCLS could trigger students’ metacognition based on the depth of cognitive processing. This result informed that SCLS increased students’ depth understanding of learning materials, based on the depth of processing model suggestions, more interaction between memory and knowledge acquisition resulted in higher academic performance. Second, it might be that teachers’ guidance in the TSDCLS could generate “safety signals,” thus easing students’ negative cognitive affect, such as decreased threatening feelings of reading anxiety. TSDCLS contributed more to students’ academic affective factors (e.g., reading anxiety) through interaction between teachers and students on problem-solving.

## Conclusion

In conclusion, both TSDCLS and SCLS contribute to the development of reading comprehension performance and development of three selected reading factors (main idea abstract, inference, and reading anxiety). Specifically, TSDCLS contributes more in reading anxiety through reducing the threatening feeling, the SCLS leads to better performance in the reading comprehension task, and both learning styles have a similar effect on inference and main idea abstraction. The TSDCLS shows a significant correlation with reading comprehension, inference, main idea abstraction, and reading anxiety, the SCLS has a significant correlation with reading comprehension, inference, and main idea abstraction, and the correlation between SCLS and reading anxiety was not significant, which needs further exploration on the compensation or interaction effect between SCLS and other reading factors. Results implicated more interaction between teacher and students on knowledge acquisition and problem-solving could decrease the threatening situation feeling which contributes more to students’ affective factors development. SCLS contributed more to students’ knowledge of deep understanding.

## Data Availability Statement

The datasets generated for this study are available on request to the corresponding author.

## Ethics Statement

The studies involving human participants were reviewed and approved by Jiaying Social Science Research Ethics Committee. Written informed consent to participate in this study was provided by the participants’ legal guardian/next of kin.

## Author Contributions

YD and SW drafted the whole paper and did data-analysis. WW and SP provided the comments and helped to revise the draft.

### Conflict of Interest

The authors declare that the research was conducted in the absence of any commercial or financial relationships that could be construed as a potential conflict of interest.
